# Successful management of Ellis III coronary perforation and complex bifurcation lesion of the right coronary artery in a patient with high-risk coronary anatomy. A case report.

**DOI:** 10.47487/apcyccv.v4i3.313

**Published:** 2023-09-30

**Authors:** Kevin Velarde-Acosta, José Raúl Delgado Arana, José M. Medina-Maguiña, Roberto Baltodano-Arellano

**Affiliations:** 1 Servicio de Cardiología Clínica. Hospital Guillermo Almenara Irigoyen - EsSalud, Lima, Perú. Servicio de Cardiología Clínica Hospital Guillermo Almenara Irigoyen - EsSalud Lima Perú; 2 Departamento de Cardiología Intervencionista. Hospital Guillermo Almenara Irigoyen - EsSalud, Lima, Perú. Departamento de Cardiología Intervencionista Hospital Guillermo Almenara Irigoyen - EsSalud Lima Perú; 3 Departamento de Imagen Cardiaca. Hospital Guillermo Almenara Irigoyen - EsSalud, Lima, Perú. Departamento de Imagen Cardiaca Hospital Guillermo Almenara Irigoyen - EsSalud Lima Perú; 4 Facultad de Medicina. Universidad Nacional Mayor de San Marcos, Lima, Perú. Universidad Nacional Mayor de San Marcos Facultad de Medicina. Universidad Nacional Mayor de San Marcos Lima Peru

**Keywords:** Percutaneous Coronary Intervention, Complications, Heart Failure, Intervención Coronaria Percutánea, Complicaciones, Insuficiencia Cardíaca

## Abstract

Complex coronary lesions represent challenging findings for percutaneous coronary intervention and potentially lead to complications during their approach. We present the case of a patient with decompensated heart failure of ischemic origin who presented multiple complex lesions on coronary angiography. The initial approach to the marginal artery was complicated by a severe coronary perforation that was satisfactorily resolved and in the second moment, a bifurcation lesion of the right coronary artery was successfully treated using the inverted Culotte technique.

## RESUMEN

Las lesiones coronarias complejas representan hallazgos desafiantes para la intervención coronaria percutánea y potencialmente exponen a complicaciones durante su abordaje. Reportamos el caso de un paciente con falla cardíaca descompensada de etiología isquémica que en la angiografía coronaria presentó múltiples lesiones complejas. El abordaje inicial de la arteria marginal se complicó con una perforación coronaria severa que se trató con éxito; en un segundo momento se abordó satisfactoriamente una lesión en bifurcación de la coronaria derecha con técnica de Culotte invertido.

## Introduction

Certain characteristics of coronary lesions have been shown to increase the complexity of percutaneous intervention (PCI). The modified ACC/AHA coronary lesion classification system defines type B2 and C lesions as complex. These lesions are associated with worse cardiovascular outcomes, increased risk of ischemic events, and increased risk of complications during PCI ^1^. We present the case of a patient with complex coronary anatomy (type C) undergoing two-stage percutaneous revascularization. Initially, angioplasty of the marginal artery was performed, and a secondary coronary perforation (Ellis III) was successfully managed; in a second stage, a bifurcated lesion in the right coronary artery was treated correctly.

## Case report

A 71-year-old male with a medical history of hypertension, diabetes mellitus, chronic kidney disease (stage III), dyslipidemia, and heart failure with reduced ejection fraction (left ventricular ejection fraction [LVEF] 20%) of ischemic etiology, came to the emergency room due to acutely decompensated heart failure with a “warm and wet” pattern. The patient was receiving medical therapy for heart failure (bisoprolol 2.5 mg qd, enalapril 5 mg bid, spironolactone 25 mg qd), in addition to aspirin 100 mg qd, atorvastatin 40 mg qd, furosemide 40 mg bid. His functional class of dyspnea was New York Heart Association (NYHA) II, Canadian Cardiovascular Society (CCS) angina class grade III, and a Kansas City Cardiomyopathy Questionnaire (KCCQ-23) score of 50.

The electrocardiogram showed sinus rhythm with signs of left atrial enlargement, left ventricular systolic overload, and poor R wave progression in precordial leads. Laboratory tests revealed an elevated pro-BNP (800 pg/mL). Chest X-ray showed cardiomegaly and signs of pulmonary congestion. Transthoracic echocardiography showed eccentric hypertrophy and global hypokinesia of the left ventricle with severe dysfunction (LVEF 20%). Right ventricular function was preserved.

**Figure 1:**
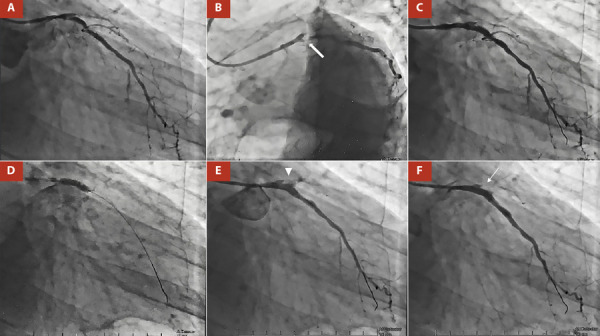
A. RAO 25°, CAU 25°: total proximal occlusion of the anterior descending artery (LAD) and circumflex artery (CX) (after the origin of the MG). B. LAO 45°, CAU 35°: obtuse marginal branch (MG) of precocious birth with a subocclusive ostial lesion (thick arrow). C. Two BioMatrix Alpha DES were implanted in the proximal in the proximal and ostial segments of the MG. Angiographic control showed under-expansion of the DES. D. Post dilatation with a 4 x 15 mm Acrostak SC balloon catheter at 11 atm E. Clear contrast extravasation (arrowhead) through an orifice of > 1 mm, making the diagnosis of an Ellis type III coronary perforation.F. Angiographic control after a covered stent BeGraft Bentley of 3.5 x 24 mm was deployed at 11 atm to close the perforation. CP was completely sealed (thin arrow).

**Figure 2:**
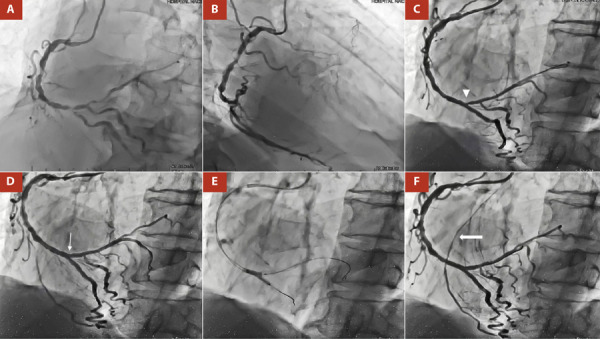
A. LAO 45°, CRA 20°: Severe stenosis of the right coronary artery (RCA) at the ostial level, middle and distal segments including its bifurcation (Medina 1-1-1). B. RAO 40°, 0°: LAD had retrograde filling from RCA, Rentrop II. C. LAO 45°, CRA 20°: Angiographic control shows impingement at the origin of the PL branch (arrowhead) after two Biomatrix DES were implanted at the level of the middle/proximal PL branch and at the distal RCA to the PD. D. POT was performed at the distal RCA level and a Resolute Onyx DES was implanted from RCA distal to PL branch. Impingement was resolved (thin arrow). E. Kissing balloon technique. F. Angiographic control showed TIMI III flow and significant improvement of heterocoronary circulation from RCA to LAD, RENTROP III (thick arrow).

After compensation, the patient was admitted to the catheterization laboratory, where severe multiarterial disease was identified **(**Figure 1A**,**1B**,**2A**,**2B**)**. Coronary angiography showed proximal occlusion of the anterior descending artery (LAD) and circumflex artery (CX) (after the origin of the obtuse marginal branch [MG]). The MG had a subocclusive ostial lesion (Video 1, Video 2) and severe stenosis of the right coronary artery (RCA) at ostial, middle and distal segments including its bifurcation (Medina 1-1-1) (Video 3, Vdeo 4). LAD had retrograde filling Rentrop II from RCA. **(**Figure 2A**,**2B; Video 4). Syntax score was 48, but the patient refused surgical revascularization.

**Figure 3:**
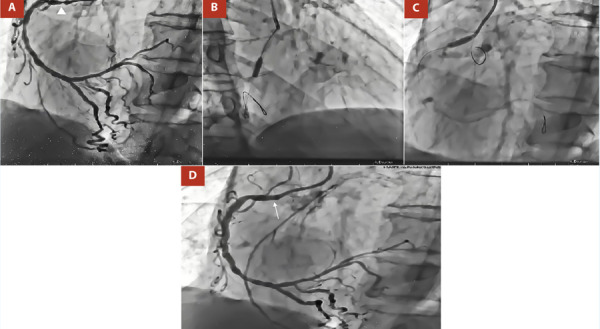
A. LAO 45°, CRA 20°: Severe stenosis of the right coronary artery (RCA) at the ostial (arrowhead) and middle segments. B, C. LAO 45°, CRA 20°: Medial and proximal RCA was predilated with NC Euphora 3.0 x 10 mm balloon at 18 atm D. Angiographic control shows impingement resolution at the ostium of RCA (thin arrow) after two Resolute Onyx DES were implanted at the level of the middle and proximal/ostial RCA using the “Floating Wire” technique.

The interventionist team decided to perform MG angioplasty. The procedure was performed via the right radial approach with a 6F introducer, using unfractionated heparin at 100 IU/kg, aiming for an activated clotting time (ACT) > 250 seconds.

After selective catheterization of the left coronary ostium with an XB 3.5 guide catheter, a 0.014-inch BMW guide wire (Abbott Vascular, Temecula) was advanced to the distal MG bed. The pre-dilatation of the proximal segment of the MG was performed using a non-compliant balloon catheter NC XPerience of 2 x 20 mm up to 12 atm, aiming for a balloon: artery ratio close to 1.

Then, a drug-eluting stent (DES) BioMatrix Alpha of 3 x 19 mm was implanted (14 atm) in the proximal segment of the MG (Video 5). A second DES BioMatrix Alpha of 3 x 14 mm was implanted (14 atm) in the ostial/proximal segment of the MG **(**Figure 1C; Video 6). The angiographic control showed under-expansion of the DES, so it was decided to post-dilate with a 4 x 15 mm Acrostak SC balloon catheter at 11 atm **(**Figure 1D; Video 7, Video 8). Control angiography after post-dilatation showed clear contrast extravasation through an orifice of >1 mm, making the diagnosis of an Ellis type III coronary perforation **(**Figure 1E; Video 9). Immediately, the perforation area was sealed by prolonged (15 min) and low pressure (5 atm) insufflation using the same balloon catheter as post-dilatation. Simultaneously, protamine (50 mg IV) was administered with the goal of an ACT < 150 seconds. Intraprocedural echocardiography showed mild pericardial effusion without hemodynamic repercussions. After waiting 15 minutes, we opted for the implantation of a polytetrafluoroethylene-coated stent, so a covered stent BeGraft Bentley of 3.5 x 24 mm was deployed (11 atm) to close the perforation. Angiographic control was performed and showed complete sealing of the coronary perforation and absence of residual lesion **(**Figure 1F; Video 10, Video 11). The patient was transferred to the Coronary Care Unit, where no intercurrences were reported.

In a second stage, revascularization of the RCA was decided. The bifurcation lesion of the distal RCA was treated using the inverted Culotte technique. The posterior descending (PD) artery, the posterolateral branch (PL), and the distal RCA were pre-dilated with a Powerline 2.0 x 15 mm balloon (8 atm) (Video 12). Next, a 2.25 x 24 mm Biomatrix DES was deployed (10 atm) at the level of the middle and proximal PL branch, and another 2.75 x 24 mm Biomatrix DES at 10 atm from the RCA distal to the PD (Video 13). Proximal optimization technique (POT) was performed at the distal RCA level with a 3.0 x 8 mm Euphora NC balloon (14 atm), a guidewire was recrossed to the PL branch, and struts were dilated in the direction of the PL branch with a 2.0 x 10 mm Euphora NC balloon at 8 atm. Another 3.0 x 8 mm Resolute Onyx DES (12 atm) was implanted from RCA distal to PL branch, and bifurcation treatment was completed with the kissing balloon technique (Video 14, Video 15, Video 16). Finally, the medial and proximal RCA were predilated with NC Euphora 3.0 x 10 mm balloon (18 atm) and 2 DES were inserted. A 3.5 x 8 mm Resolute Onyx DES (14 atm) was positioned at the middle segment, and a 3.5 x 8 mm Resolute Onyx DES (16 atm) for the proximal/ostial segment using the “Floating Wire” technique. Angiographic control showed TIMI III flow and a significant improvement of heterocoronary circulation from RCA to LAD, RENTROP III (Video 17, Video 18).

## Discussion

Coronary artery perforation (CP) is a rare complication but potentially fatal if not treated promptly. The incidence of this complication ranges from 0.43% to 2.9%, being directly proportional to the complexity of the procedure ^2^. The mortality rate depends on the severity of the CP and can reach up to 21.2% ^2^. Several factors associated with an increased risk of coronary perforation have been described. These include anatomical factors (complex coronary lesions type B2, C; severe calcification, tortuosity, chronic total occlusion) and procedural factors (use of Hydrophilic-coated, polymer jacketed, and stiff-tip guidewires, aggressive balloon inflation or DES, use of atheroablative devices) ^3^^,^^4^.

CPs are stratified using the Ellis Classification ^4^. There are 3 types: Type I: Presence of extraluminal crater without extravasation; Type II: Presence of pericardial or myocardial blush without contrast jet extravasation; Type III: Presence of contrast jet extravasation through frank perforation (≥1 mm); and, Type IV: Presence of contrast jet extravasation into the cavities like a cardiac chamber or coronary sinus.

After CP, depending on the severity, patients may develop pericardial tamponade, cardiogenic shock, myocardial infarction, and even death if not managed promptly. The management of perforations will depend on their severity and also on their location. In general, Ellis I and II perforations can be managed conservatively, with prolonged balloon inflation prior to the point of contrast extravasation ^5^. On the other hand, more severe perforations usually require additional management, either with covered stents, coils, microspheres, autologous blood clots, or autologous subcutaneous fat or surgical management ^5^.

In the case presented, the patient had several risk factors for the development of perforation and after aggressive post-dilatation, the patient developed an Ellis III type CP. Since it was a severe perforation, located in the middle segment of the vessel, reversal of anticoagulation, extended balloon inflation prior to the perforated segment (15 min), and the implantation of a covered stent were chosen, with adequate control of the complication. Due to the rapid action, pericardial effusion was mild and there was no hemodynamic compromise of the right cardiac cavities.

On the other hand, coronary bifurcation lesions (CBL) represent one of the most challenging treatments in interventional cardiology. They account for approximately 15-20% of all PCIs ^6^. For more than 15 years, the technique of choice for the management of CBL has been the provisional stenting technique (PST); however, over time, the concepts of complex CBL have been developed and, likewise, various double stenting techniques (DST) have been perfected. Thus, the recently published DEFINITION II trial established the criteria for defining complex CBL **(**Table 1**)** and objectively demonstrated the reduction in the rate of target vessel failure (mainly due to a reduction in the target vessel MI and clinically driven target lesion revascularization rate) when comparing DST vs PST strategies ^7^.

**Table 1:** Definition criteria for a complex coronary bifurcation lesion

Major criteria	Minor criteria	CBL definition
For left main bifurcation (Major 1) SB lesion length ≥ 10 mm and SB diameter stenosis ≥ 70%	> Mild calcification	Major 1 or Major 2 + any 2 minor criteria
	Multiple lesions	
	Bifurcation angle < 45° or > 70°	
For non-left main bifurcation (Major 2) SB lesion length ≥ 10 mm and SB diameter stenosis ≥ 90%	MV-RVD < 2.5 mm	
	MV lesion length ≥ 25 mm	
Thrombus-containing lesions

All bifurcation lesions are true CBL (Medina 1-1-1 or 0-1-1). CBL: coronary bifurcation lesion; MV: main vessel; RVD: reference vessel diameter; SB: side branch.

Our patient presented, a CBL (Medina 1-1-1) at the distal RCA level (proximal main vessel), with a side branch with a proximal lesion of > 10 mm, stenosis > 90%, a bifurcation angle of 40° and multiple lesions in the distal segment of the SB, thus fulfilling the criteria for complex CBL. For this reason, a DST was performed. Although, in DEFINITION II trial ^7^, the most commonly used DST was double kissing (DK)-crush (77.8%), in our patient the inverted Culotte stenting technique was chosen. This was mainly due to the fact that the operator’s familiarity with the technique to be performed is fundamental for the success of the procedure.

The angiographic result of the procedure was excellent, obtaining a TIMI III flow, residual stenosis < 20%, and a significant improvement in the heterocoronary circulation (RENTROP III). It should be noted that the patient had chronic total occlusion of the LAD and CX, which is essential to optimize blood flow from the RCA to the LAD. Although the REVIVED BCIS 2 trial ^8^, has demonstrated the lack of benefit of multivessel PCI in patients with reduced LVEF heart failure and extensive coronary artery disease, studies with a larger population and longer prospective follow-up are still pending. Despite this, the patient had a favorable outcome and is currently being monitored on an outpatient basis, tolerating optimal medical therapy (bisoprolol 5 mg qd, losartan 50 mg bid, spironolactone 50 mg qd, dapaglifozin 10 mg qd), reports improvement in his functional (NYHA I, CCS II and KCCQ-23 score was 80). There has been no other hospitalization for decompensated acute failure.

In conclusion, complex coronary lesions (type B2, C), as defined by the ACC/AHA, are associated with worse cardiovascular outcomes and a higher risk of complications during PCI. CP is one of these complications that, although rare, is potentially lethal, and its timely identification and optimal management with therapeutic strategies based on the severity and location of the perforation is essential. CBL are part of the complex coronary lesions and are one of the most challenging therapeutic challenges for interventional cardiologists. It is essential to determine whether the patient meets the criteria for complex CBL and, based on this, to determine the revascularization strategy.
